# Transcriptome analysis of rice root heterosis by RNA-Seq

**DOI:** 10.1186/1471-2164-14-19

**Published:** 2013-01-16

**Authors:** Rongrong Zhai, Yue Feng, Huimin Wang, Xiaodeng Zhan, Xihong Shen, Weiming Wu, Yingxin Zhang, Daibo Chen, Gaoxing Dai, Zhanlie Yang, Liyong Cao, Shihua Cheng

**Affiliations:** 1 State Key Laboratory of Rice Biology, China National Rice Research Institute, Hangzhou, 310006, China; 2 College of Agronomy, Shenyang Agricultural University, Shenyang, 110161, China; 3 Rice Research Institute, Guangxi Academy of Agricultural Sciences, Nanning, 530007, China; 4 Rice Research Institute, Guizhou Academy of Agricultural Sciences, Guiyang, 550006, China

**Keywords:** Heterosis, Root, Transcriptome, RNA-Seq, Hybrid rice

## Abstract

**Background:**

Heterosis is a phenomenon in which hybrids exhibit superior performance relative to parental phenotypes. In addition to the heterosis of above-ground agronomic traits on which most existing studies have focused, root heterosis is also an indispensable component of heterosis in the entire plant and of major importance to plant breeding. Consequently, systematic investigations of root heterosis, particularly in reproductive-stage rice, are needed. The recent advent of RNA sequencing technology (RNA-Seq) provides an opportunity to conduct in-depth transcript profiling for heterosis studies.

**Results:**

Using the Illumina HiSeq 2000 platform, the root transcriptomes of the super-hybrid rice variety Xieyou 9308 and its parents were analyzed at tillering and heading stages. Approximately 391 million high-quality paired-end reads (100-bp in size) were generated and aligned against the Nipponbare reference genome. We found that 38,872 of 42,081 (92.4%) annotated transcripts were represented by at least one sequence read. A total of 829 and 4186 transcripts that were differentially expressed between the hybrid and its parents (DG_HP_) were identified at tillering and heading stages, respectively. Out of the DG_HP_, 66.59% were down-regulated at the tillering stage and 64.41% were up-regulated at the heading stage. At the heading stage, the DG_HP_ were significantly enriched in pathways related to processes such as carbohydrate metabolism and plant hormone signal transduction, with most of the key genes that are involved in the two pathways being up-regulated in the hybrid. Several significant DG_HP_ that could be mapped to quantitative trait loci (QTLs) for yield and root traits are also involved in carbohydrate metabolism and plant hormone signal transduction pathways.

**Conclusions:**

An extensive transcriptome dataset was obtained by RNA-Seq, giving a comprehensive overview of the root transcriptomes at tillering and heading stages in a heterotic rice cross and providing a useful resource for the rice research community. Using comparative transcriptome analysis, we detected DG_HP_ and identified a group of potential candidate transcripts. The changes in the expression of the candidate transcripts may lay a foundation for future studies on molecular mechanisms underlying root heterosis.

## Background

Heterosis is a phenomenon in which hybrids exhibit superior phenotypes, such as enhanced biomass production, development rate, grain yield, and stress tolerance, relative to their parents. Heterosis has been effectively utilized to increase crop production in the world. It is estimated, for example, that hybrid rice, which occupies more than 50% of the total rice area in China, has a 10–20% yield advantage over inbred varieties [1]. Our knowledge of the genetic mechanisms of heterosis, however, has lagged behind its wide application. Two hypotheses-dominance [2,3] and over-dominance [4,5]-were proposed in the early 20th century to interpret heterosis. Both describe nonadditive behavior as a consequence of genetic differences between distinct homozygous parental lines and their heterozygous hybrids [6]. With the advent of molecular makers, quantitative trait locus (QTL) mapping has become a routine tool for studying the genetic basis of heterosis in crop plants; so far, however, the QTL analysis has not contributed much to the understanding of heterosis.

With the development of functional genomics, the technique of large-scale transcriptome analysis-based on cDNA or expressed sequence tag (EST) library sequencing, microarray hybridization, and serial analysis of gene expression (SAGE)-has been used to investigate heterosis in *Arabidopsis*[7,8], maize [9], and rice [10-12]. Such technologies offer the potential to unveil the molecular basis of heterosis at the transcriptional level [13,14]. These technologies have drawbacks, however, such as low throughput, high cost, low sensitivity, cloning bias, high background signal, and pre-determined probes requirements [15]. Next-generation high-throughput RNA sequencing technology (RNA-Seq) is a recently-developed method for discovering, profiling, and quantifying RNA transcripts with several advantages over other expression profiling technologies including higher sensitivity and the ability to detect splicing isoforms and somatic mutations [16]. Using RNA-Seq, significant progress has been made in understanding the transcript expression of rice over the last two years [15,17-21]. For example, transcriptome analysis of rice mature root tissue and root tips at two time points identified 1761 root-enriched transcripts and 306 tip-enriched transcripts involved in different physiological processes [15]. In addition, RNA-Seq has been applied to the identification of stress-inducible transcripts in rice [17,21]. Other than a transcriptome analysis of seedling shoots at the four-leaf stage [18], however, little effort is being expended in attempts to investigate heterosis using RNA-Seq.

Plant root systems serve a number of important functions, including anchoring the plant, absorbing water and nutrients, producing amino acids and hormones, and secreting organic acids, enzymes, and alkaloids. In recent years, considerable research and interest has focused on root systems. Some of these studies have demonstrated that heterosis levels might be higher in root traits than in aboveground agronomic traits [22-24], which suggests that roots might be an ideal organ for investigating the genetic basis of rice heterosis. Several attempts have been made to discover the molecular mechanism of root heterosis at the vegetative stage [25-27], but little attention has been paid to heterosis in the root system during the late growth stage, when accumulation, transportation, and distribution of dry matter, and ultimately, yield potential, may be influenced. There has currently been no systematic investigation into root heterosis at the two different developmental stages.

In this study, we focused our heterosis research on the late-stage high-vigor super-hybrid rice variety, Xieyou 9308, which has a grain yield of up to 12.23 × 10^3^ kg ⋅ hm^-2^[28] and was designated as “super rice” by the Chinese Ministry of Agriculture in 2005. Xieyou 9308 was bred by crossing the restorer line R9308 (with 25% *japonica* genetic components) to the maternal line Xieqingzao B (*indica*). We used RNA-Seq to investigate the global transcriptomes of roots from Xieyou 9308 and its parents at tillering and heading stages. Differentially expressed transcripts and their expression patterns were analyzed, and several potential candidate transcripts were found to be involved in carbohydrate metabolism and plant hormone signal transduction pathways. We expect this genome-wide transcriptome comparison to provide a starting point to understand the causative mechanism of the altered gene expression in the hybrid and the molecular mechanism underlying rice root heterosis.

## Results

### Characterization of Xieyou 9308 and its two parental lines

**Figure 1:**
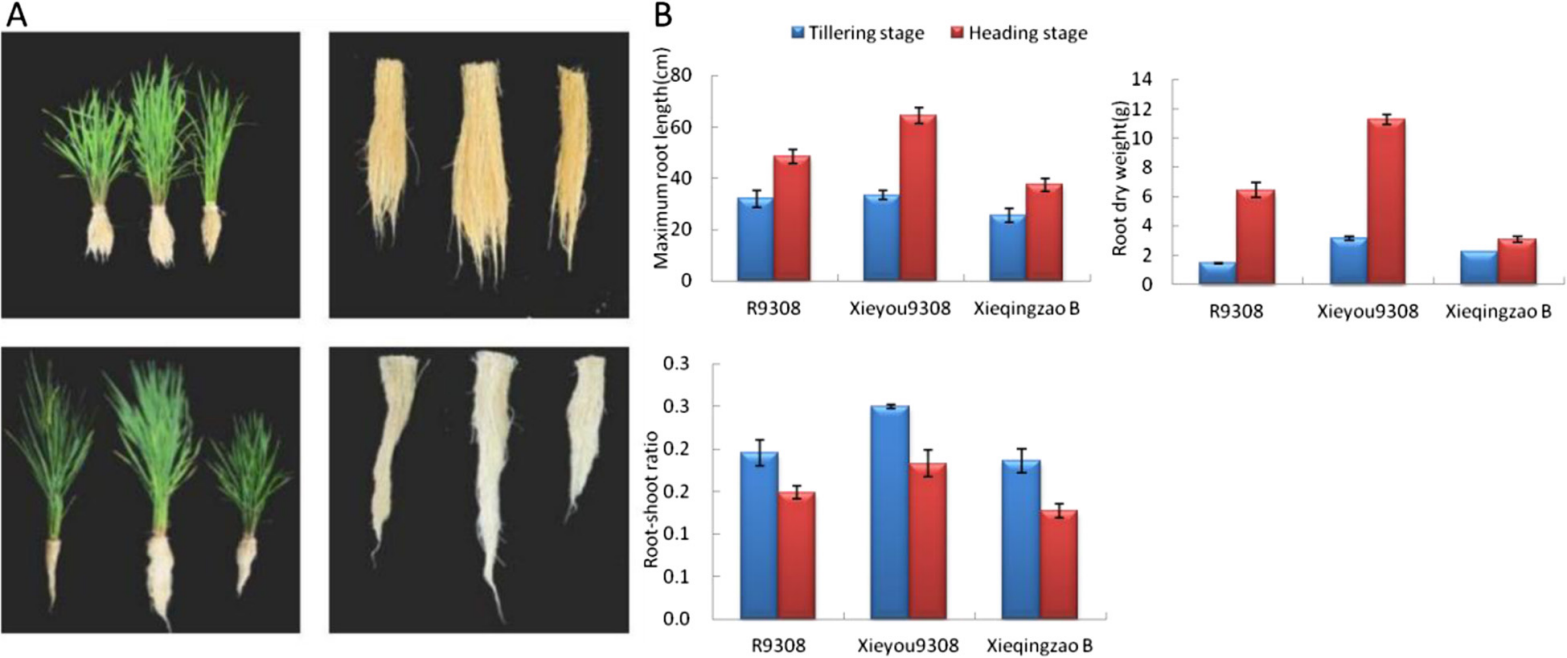
**Comparisons of heterosis in the super-hybrid rice combination Xieyou 9308.** (**A**) The upper panel illustrates the cross between Xieqingzao B and R9308 at the tillering stage. Left, Xieqingzao B; middle, Xieyou 9308; right, R9308. The lower panel shows the cross at the heading stage. Left, R9308; middle, Xieyou 9308; right, Xieqingzao B. (**B**) Root traits of Xieyou 9308, Xieqingzao B, and R9308 at tillering and heading stages.

We used RNA-Seq to investigate gene expression and function in a heterotic cross involving Xieyou 9308, a super-hybrid rice variety with superior yield performance, its maternal line Xieqingzao B, and its paternal line R9308. As suggested by Pickett [29], other yield-related traits might also show heterosis. In this study, we found that the roots and aerial parts of Xieyou 9308 were more vigorous than those of either parent (Figure 1). Mid-parent heterosis (MPH) and best-parent heterosis (HPH) were calculated to measure the heterosis of aerial parts and roots (see Methods). The quantification of MPH and HPH and their statistical significance were shown in Table 1. We observed significant MPH (*p* < 0.05) for all traits at both stages except for shoot dry weight at the tillering stage. Furthermore, we also observed significant HPH (*p* < 0.05) for the root-shoot ratio at the tillering stage and root length, root dry weight, and shoot dry weight at the heading stage. The MPH and HPH of root dry weight were greater than those of shoot dry weight at both stages, indicating that the level of heterosis was much higher in roots than in aerial parts. At the tillering stage, the MPH of root length, root dry weight, and root-shoot ratio varied from 15.88% to 68.56%. By comparison, the degree of heterosis for these traits was larger at the heading stage, with the MPH ranging from 28.57% to 137.26%.

**Table 1:** Mid-parent heterosis and high-parent heterosis of root traits at tillering and heading stages

**Stage**	**Traits**	**R9308**	**Xieyou 9308**	**Xieqingzao B**	**MPH (%)**	**HPH (%)**
Tillering stage	Root length (cm)	31.96±3.21	33.34±1.77	25.58±2.63	15.88^**^	4.32
	Root dry weight (g)	1.45±0.04	3.11±0.13	2.24±0.01	68.56^*^	38.84
	Root-shoot ratio	0.20±0.02	0.25±0.00	0.19±0.01	28.21^*^	25.00^*^
	Shoot dry weight (g)	7.45±0.81	12.48±0.39	12.07±0.85	27.85	3.36
Heading stage	Root length (cm)	48.63±2.73	64.45±3.07	37.5±2.45	49.66^**^	32.53^**^
	Root dry weight (g)	6.44±0.52	11.27±0.34	3.06±0.22	137.26^**^	75.00^**^
	Root-shoot ratio	0.15±0.00	0.18±0.02	0.13±0.00	28.57^*^	20.00
	Shoot dry weight (g)	43.21±1.34	61.86±0.64	24.13±5.15	92.29^*^	43.16^*^

^**^Significant difference with *p* < 0.01.

^*^Significant difference with *p* < 0.05.

### Mapping reads to the rice genome

To analyze the transcriptomes of the above three genotypes at tillering and heading stages, cDNA libraries were prepared from rice roots and subjected to RNA-Seq analysis on the Illumina HiSeq 2000 platform. In total, 448 million short reads were generated from the two developmental stages, with 391 million high-quality 100-bp reads selected for further analysis (see Methods). The two biological replicates were in good agreement with respect to gene expression levels, with 0.86 <*R*^2^ < 0.96 (Additional file 1: Figure S1). We pooled the short reads and aligned them against the Nipponbare reference genome (IRGSP build 5.0); 50.32–73.09% of reads were mapped to exonic regions, 2.12–2.83% to intronic regions, and 4.04–5.66% to intergenic regions (Table 2). We also found that 38,872 of 42,081 (92.4%) annotated transcripts in the Nipponbare reference genome were detected by at least one sequence read.

**Table 2:** Number of mapped reads

**Sample**	**Total filtered reads**	**Exon**	**%**	**Intron**	**%**	**Intergenenic**	**%**
F12	75,780,826	40,030,725	52.82	1,609,267	2.12	3,060,859	4.04
R12	71,254,122	52,078,194	73.09	1,796,654	2.52	3,890,885	5.46
X12	79,939,758	40,222,370	50.32	1,745,087	2.18	3,398,300	4.25
F34	50,520,686	36,079,475	71.42	1,301,039	2.58	2,746,963	5.44
R34	52,582,652	37,388,453	71.10	1,487,063	2.83	2,974,902	5.66
X34	61,330,602	43,101,722	70.28	1,533,592	2.50	3,273,276	5.34
Total	391,408,646	248,900,939	63.59	9,472,702	2.42	19,345,185	4.94

R, X, and F denote R9308, Xieqingzao B, and Xieyou 9308, respectively. Numbers 12 and 34 denote samples from roots at tillering and heading stages, respectively.

### Transcriptome profiles of Xieyou 9308 and its parents

**Figure 2:**
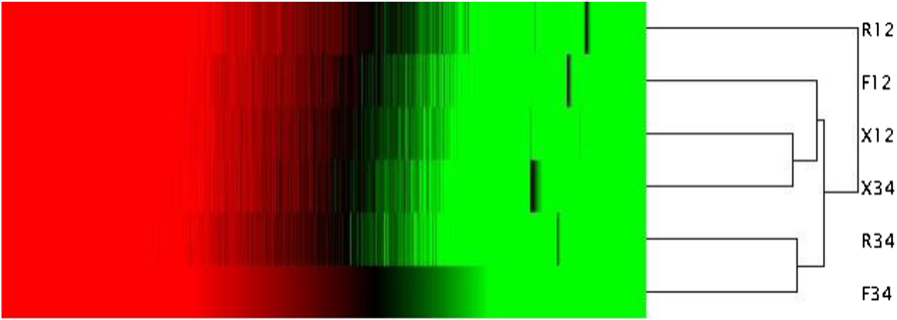
**Hierarchical cluster analysis of all transcripts.** The color key represents RPKM normalized log_2_ transformed counts. R, X, and F denote R9308, Xieqingzao B, and Xieyou 9308, respectively. Numbers 12 and 34 denote samples from roots at tillering and heading stages, respectively.

Correlations between the hybrid and its parents at tillering and heading stages were investigated using cluster analysis with Cluster 3.0 software. Xieqingzao B had smaller differences in gene expression between the two stages than did R9308 and Xieyou 9308 (Figure 2). Interestingly, with respect to root traits such as root dry weight, there were larger differences between the two stages in R9308 and Xieyou 9308 than in Xieqingzao B (Additional file 2: Figure S2). In addition, the transcriptome profile of Xieyou 9308 was similar to Xieqingzao B (maternal) at the tillering stage, but it was closer to R9308 (paternal) at the heading stage (Figure 2). This is consistent with the results obtained from root dry weight at corresponding stages (Table 1).

### Identification of differentially expressed genes (DEGs) by RNA-Seq

**Figure 3:**
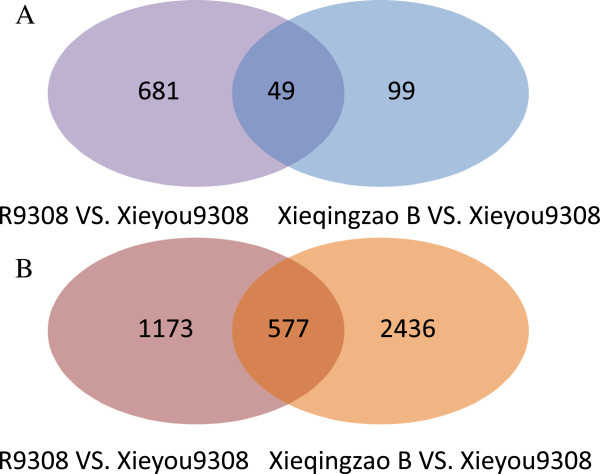
**DEGs in the rice heterotic crosses.** Venn diagram of DEGs between the hybrid and its parents at (**A**) tillering and (**B**) heading stages.

Gene expression levels were measured as reads per kilobase per million reads (RPKM), with RPKM values ranging from 0 to over 10^4^. Putative DEGs were identified using the following criteria: (1) false discovery rate (FDR) less than or equal to 0.05 and (2) fold change (FC) greater than or equal to 2. Using these criteria, we identified 1776 transcripts as reliable DEGs at the tillering stage and 4991 transcripts at the heading stage among the three genotypes. For a detailed comparison, see Additional file 3: Table S1. DEGs between the hybrid and its parents are designated as DG_HP_, and those between the parental lines are designated as DG_PP_. DG_HP_ may be relevant to heterosis because differences in expression between the hybrid and its parents should underlie their phenotypic differences, while DG_PP_ only represents the differences between the two parental lines [12]. In total, 829 and 4186 DG_HP_ were identified at tillering and heading stages, respectively (Table 3, Figure 3A and B). Of the 829 DG_HP_ at the tillering stage, 730 transcripts showed differences between R9308 and Xieyou 9308, whereas only 148 exhibited differences between Xieqingzao B and Xieyou 9308. At the heading stage, 3013 transcripts showed differences between Xieqingzao B and Xieyou 9308, whereas only 1750 transcripts exhibited differences between R9308 and Xieyou 9308 (Table 3). These results suggest that gene expression in Xieyou 9308 is more similar to that in Xieqingzao B than to that in R9308 at the tillering stage. In contrast, at the heading stage, gene expression in Xieyou 9308 is more similar to that in R9308 than to that in Xieqingzao B. These observations are consistent with the results from the hierarchical cluster analysis described above.

**Table 3:** Number and classification of DEGs

**Stage**	**DG**_**PP**_	**DG**_**HP**_	**R/F**	**X/F**	**Total**
Tillering stage	1465	829	730	148	1776
Heading stage	2971	4186	1750	3013	4991

R, X, and F refer to R9308, Xieqingzao B, and Xieyou 9308, respectively. DG_PP_ refers to DEGs between both parents, and DG_HP_ refers to DEGs between the hybrid and parents.

**Figure 4:**
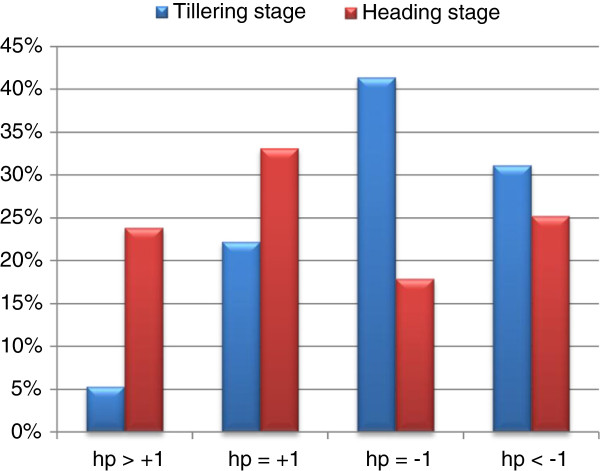
**Breakdown of the DG**_**HP**_**at tillering and heading stages according to the dominance ratio *h***_**p**_**.** For the differences [*d*] - [*a*] ([*d*] >0) and [*d*] + [*a*] ([*d*] <0), 99.8% confidence intervals were constructed. Depending on the sign of [*d*], *h*_p_ was classified as either positive or negative.

Treating gene expression levels as quantitative traits, traditional quantitative genetic parameters, such as composite additive effect [*a*] and composite dominance effect [*d*], were estimated for our expression profile. We classified DG_HP_ according to the dominance ratio *h*_p_ (= [*d*]/[*a*]), based on 99.8% confidence intervals constructed for [*d*] - [*a*] ([*d*] > 0) and [*d*] + [*a*] ([*d*] < 0) (see Methods). Depending on the sign of [*d*], *h*_p_ was classified as either negative or positive (Additional file 4: Table S2). DG_HP_ were then classified into four expression patterns: above high-parent (AHP) (*h*_p_ > 1), high-parent level (HPL) (*h*_p_ = 1), low-parent level (LPL) (*h*_p_ = −1), and below low-parent (BLP) (*h*_p_ < −1). As shown in Figure 4, transcripts that were classified as LPL at the tillering stage and transcripts that were classified as HPL at the heading stage accounted for most of the DG_HP_. In addition, there were more down-regulated transcripts (LPL and BLP) than up-regulated transcripts (AHP and HPL) at the tillering stage. At the heading stage, however, the results were completely reversed.

### Functional classification by Gene Ontology (GO)

**Figure 5:**
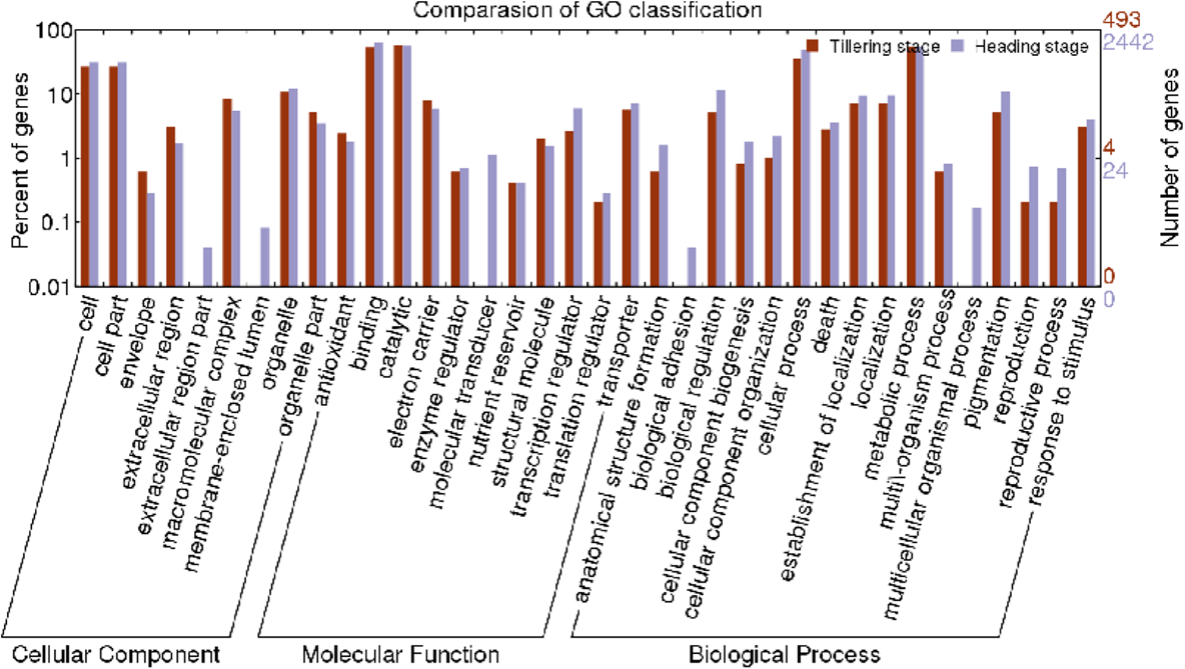
**Comparison of Gene Ontology (GO) classifications of DG**_**HP**_**at tillering and heading stages.**

GO slim was used for the functional classification of DG_HP_, and the annotations were plotted using Web Gene Ontology Annotation Plot (WEGO) software [30]. In total, 493 of the 829 DG_HP_ at the tillering stage and 2442 of the 4186 DG_HP_ at the heading stage were assigned to at least one term in GO molecular function, cellular component, and biological process categories. Transcripts from the two stages were further classified into 42 functional subcategories, providing an overview of ontology content (Figure 5). In the biological process category, cellular process and metabolic process were the most highly represented groups, indicating that extensive metabolic activities were taking place in roots of hybrid plants during both stages. In the molecular function category, binding and catalytic processes were prominently represented, while cell and cell parts dominated the cellular component category.

We further identified GO terms in the biological process category that were over-represented (*p* < 0.05) in DG_HP_ at tillering and heading stages (Tables 4 and 5). These GO terms served as indicators of significantly different biological processes underlying heterosis at tillering and heading stages. GO terms such as metabolic process, carbohydrate metabolic process, oxidation reduction, photosynthesis (light harvesting), photosynthesis, apoptosis, and response to oxidative stress were enriched in both sets of transcripts from the two stages, suggesting that the same biological processes were required to maintain root activities during both tillering and heading stages. Some striking differences were found, however, between the two sets of enriched GO terms. In particular, GO terms related to protein phosphorylation, transport, cellulose biosynthetic process, and glycolysis were highly enriched in DG_HP_ at the heading stage (Additional file 5: Figure S3).

**Table 4:** **Significant GO terms of DG**_**HP**_**in the biological process category at the tillering stage**

**GO_term**	**GO_term_annotation**	***p***
GO:0008152	metabolic process	2.16E-10
GO:0005975	carbohydrate metabolic process	0.000185
GO:0055114	oxidation reduction	1.20E-08
GO:0009765	photosynthesis, light harvesting	8.77E-28
GO:0015979	photosynthesis	4.18E-15
GO:0006915	apoptosis	0.029295
GO:0006979	response to oxidative stress	0.046569
GO:0055085	transmembrane transport	0.006749
GO:0006032	chitin catabolic process	0.006834
GO:0016998	cell wall macromolecule catabolic process	0.033934
GO:0015977	carbon fixation	4.81E-05
GO:0006098	pentose-phosphate shunt	0.003488
GO:0006952	defense response	0.025179
GO:0006006	glucose metabolic process	0.020128
GO:0006808	regulation of nitrogen utilization	0.033911
GO:0009607	response to biotic stimulus	0.003078
GO:0022900	electron transport chain	0.010189
GO:0016114	terpenoid biosynthetic process	0.009143
GO:0050832	defense response to fungus	0.003784
GO:0016559	peroxisome fission	0.020856
GO:0019509	L-methionine salvage from methylthioadenosine	0.016498
GO:0015995	chlorophyll biosynthetic process	0.04225
GO:0042742	defense response to bacterium	0.003784

**Table 5:** **The top 20 most represented GO terms of DG**_**HP**_**in the biological process category at the heading stage**

**GO_term**	**GO_term_annotation**	***p***
GO:0008152	metabolic process	0.001293
GO:0006468	protein phosphorylation	1.82E-06
GO:0045449	regulation of transcription	0.024656
GO:0005975	carbohydrate metabolic process	0.004871
GO:0006810	transport	0.001456
GO:0006915	apoptosis	2.43E-08
GO:0055114	oxidation reduction	0.000697
GO:0006979	response to oxidative stress	0.026271
GO:0030244	cellulose biosynthetic process	4.83E-10
GO:0044237	cellular metabolic process	0.011942
GO:0015979	photosynthesis	1.43E-06
GO:0006857	oligopeptide transport	0.011219
GO:0006096	glycolysis	0.046925
GO:0009765	photosynthesis, light harvesting	1.16E-09
GO:0006334	nucleosome assembly	0.007926
GO:0007018	microtubule-based movement	0.008653
GO:0048544	recognition of pollen	0.032814
GO:0006855	drug transmembrane transport	0.02111
GO:0007017	microtubule-based process	0.000615
GO:0051258	protein polymerization	6.73E-05

### Kyoto Encyclopedia of Genes and Genomes (KEGG) pathway mapping

**Figure 6:**
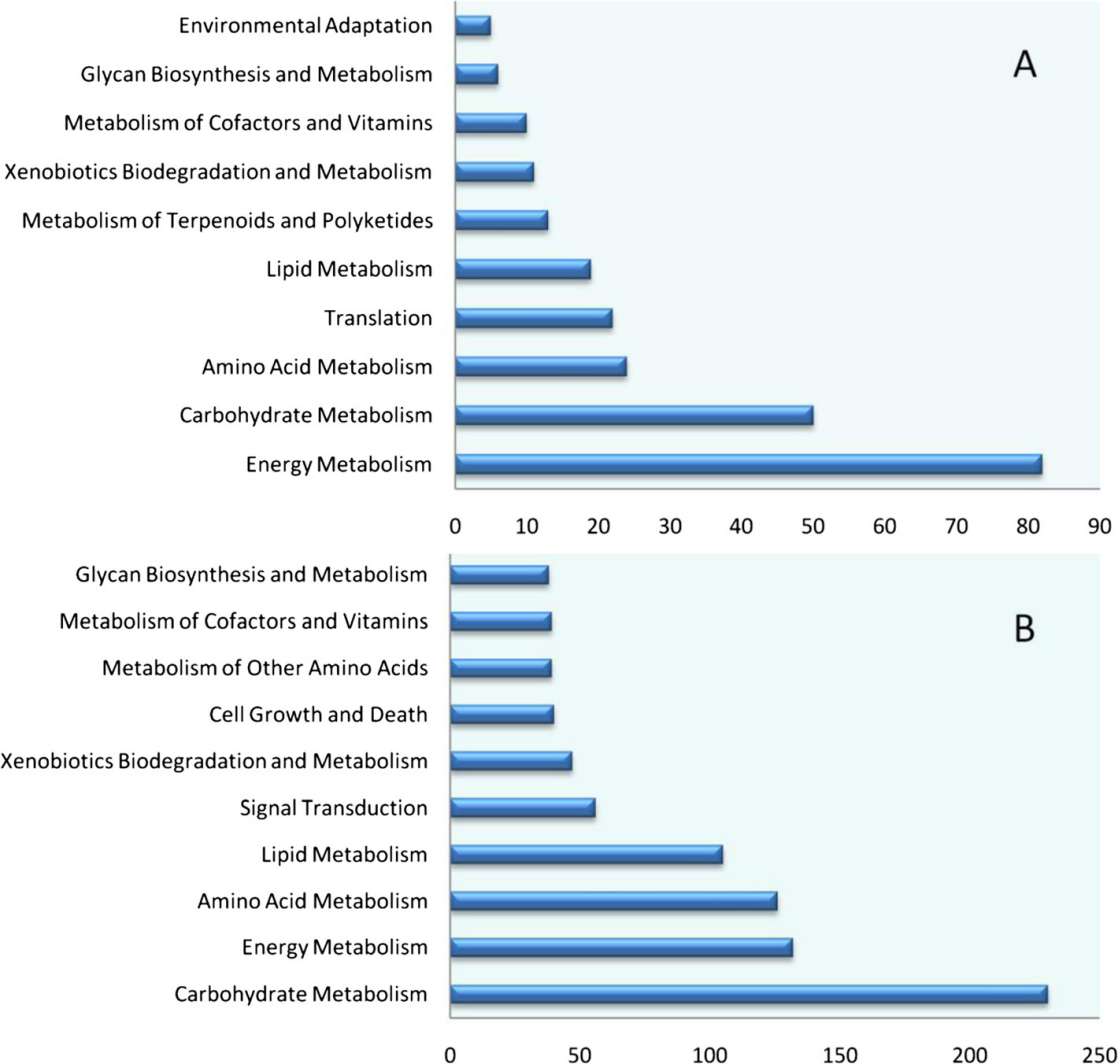
**KEGG pathway assignments at (A) tillering and (B) heading stages.** The top 10 most represented categories and the number of transcripts predicted to belong to each category are shown.

To identify metabolic pathways in which DG_HP_ were involved and enriched, pathway-based analysis was performed using the KEGG pathway database. As a result, 200 of 829 DG_HP_ at the tillering stage and 830 of 4186 DG_HP_ at the heading stage were classified into 12 functional categories. As shown in Figure 6, these transcripts belonged mainly to the following KEGG pathways at both stages: carbohydrate metabolism, energy metabolism, amino acid metabolism, and lipid metabolism.

The above DG_HP_ were then classified into 164 subcategories that corresponded to their functions. We further identified KEGG Orthology (KO) terms that were over-represented in the DG_HP_ (Additional file 6: Table S3 and Additional file 6: Table S4). Carbon fixation, photosynthesis, photosynthesis-antenna protein pathways, and fructose and mannose metabolism were over-represented at both stages. In contrast, KO terms related to signal transduction, glycolysis/gluconeogenesis, amino sugar and nucleotide sugar metabolism, and starch and sucrose metabolism were highly enriched in DG_HP_ and were exclusively expressed at the heading stage (Additional file 7: Figure S4). This suggests that there are considerable differences in root physiological processes between the tillering stage and the heading stage. These annotations provide a valuable resource for investigating specific processes, functions, and pathways underlying heterosis.

### Validation by quantitative real-time PCR (qRT-PCR)

**Figure 7:**
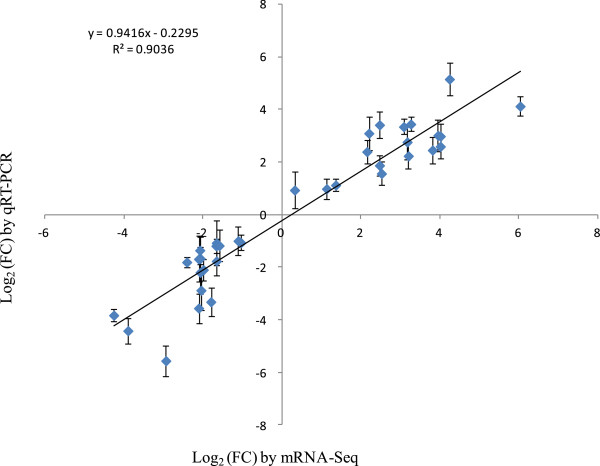
**Comparison of the log**_**2**_**(FC) of 18 selected transcripts using RNA-Seq and qRT-PCR.**

A subset of 18 DG_HP_ was selected for qRT-PCR validation at tillering and heading stages. qRT-PCR primers were designed based on Rice Annotation Project Database (RAP-DB) annotations, and their sequences were listed in Additional file 8: Table S5. We compared the results obtained from qRT-PCR with those generated from RNA-Seq analysis of these transcripts. Expression trends were consistent for all transcripts in both analyses, with a correlation coefficient of *R*^2^ = 0.9036 (Figure 7).

## Discussion

Although heterosis has been widely exploited in plant breeding and plays an important role in agriculture, the molecular and genetic mechanisms underlying the phenomenon remain poorly understood. Differential gene expression between a hybrid and its parents may be associated with heterosis [10-12,18,31]. In this study, we used RNA-Seq to investigate the relationship between transcriptional profiles and heterosis in a super-hybrid rice combination, Xieyou 9308. In our RNA-Seq analysis, 391 million high-quality 100-bp paired-end reads were generated from the roots of Xieyou 9308 and its parental lines at tillering and heading stages, and 38,872 annotated transcripts were identified. On average, 9301 reads were detected per identified annotated transcript, providing approximately 70-fold coverage of the transcriptome. From the annotated transcripts, 829 DG_HP_ at the tillering stage and 4186 DG_HP_ at the heading stage were identified. These results suggest that the expression of DG_HP_ at the heading stage may play a more important role in root heterosis than that at the tillering stage. Additionally, only a small fraction of transcripts may be responsible for root heterosis at the transcriptional level in Xieyou 9308.

### Comparative analysis of annotated DG_HP_

Comparative transcriptome analysis revealed a subset of transcripts that were differently expressed between the hybrid and its parents at tillering and heading stages. Some potential regulators for heterosis in root development were uncovered. At the tillering stage, a large number of DG_HP_ related to carbon fixation in photosynthetic organisms, photosynthesis, and photosynthesis-antenna proteins were found. Transcripts involved in plant hormone signal transduction, carbon fixation in photosynthetic organisms, photosynthesis, photosynthesis-antenna proteins, and carbohydrate metabolism (including glycolysis and starch/sucrose, fructose/mannose, glucose/galactose, and pyruvate metabolism) were highly expressed in roots at the heading stage. We therefore conclude that carbohydrate metabolism and plant hormone signal transduction pathways may contribute significantly to root development. Another result of interest is the differential expression of photosynthetic transcripts at both stages. The observed gene expression may be related to culture effects because the expression of these transcripts is not generally observed in roots of soil-grown plants [32]. In our study, plants were cultured under hydroponic conditions; the roots may have thus been passively exposed to light, which could strongly activate photosynthesis in root tissues. A similar result was observed in another recent study, along with the finding that a large number of DEGs were involved in photosynthesis in roots [15]. In this study, we found that most of the DG_HP_ were down-regulated at the tillering stage and up-regulated at the heading stage. Because Xieyou 9308 is a late-stage high-vigor super-hybrid rice variety, root heterosis might be expected to be stronger at the heading stage than at the tillering stage. For these reasons, subsequent analyses that focus on the expression of DG_HP_ at the heading stage are warranted.

### The role of carbohydrate metabolism in heterosis

**Figure 8:**
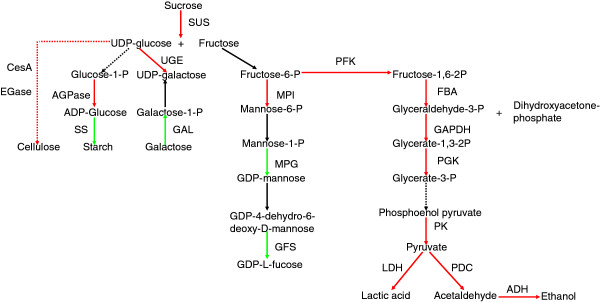
**Predicted carbohydrate pathways in rice roots.** Red arrows indicate transcripts that were up-regulated in Xieyou 9308 compared with the parental lines. Green arrows indicate down-regulated transcripts. Black indicates no change in gene expression levels.

Carbohydrate metabolism is an essential process in plants that produces both energy sources and structural components of cells and cell walls [33]. It also generates compatible solutes for osmotic adjustment in roots [34-36]. In our study, significant differences in carbohydrate metabolism, including glycolysis and metabolism of starch/sucrose, fructose/mannose, glucose/galactose, and pyruvate, were detected in the root tissues of the hybrid and its parents. This result is consistent with the fact that a variety of carbohydrates are present in root tissues and exudates [37]. Many of the DG_HP_-encoded enzymes involved in carbohydrate metabolism belong to a complex network that regulates carbohydrate synthesis/turnover in roots (Figure 8). The starting point for the carbohydrate synthetic pathway in root tissues is sucrose, which is the main form of photosynthate that is transported from shoots to roots. Ogawa *et al.*[38] determined that sucrose is transported to the root elongation zone and the surrounding tissue of the lateral root primordia, where it is converted to hexose by invertase or sucrose synthase (SUS). Hexose serves as an energy source, a compatible solute for root system formation, and a substance for cell wall synthesis. In this regard, transcripts encoding SUS (Os03t0401300, *SUS1*; Os04t0249500, *OsSUS7*; Os03t0340500, *SUS4*) were up-regulated in Xieyou 9308 compared with the parental lines. As reported previously, *OsSUS7* is highly expressed in roots [39], and *SUS1* is predominantly expressed in elongating tissues, including roots where rapid formation of secondary wall takes place just after cell elongation [40]. The high level of SUS expression in Xieyou 9308 may play a major role in sink organs by providing carbon sources for sink metabolism and SUS expression may promote root elongation.

Some enzymes involved in glycolysis pathways, such as phosphofructose kinase (PFK), fructose-bisphosphate aldolase (FBA), glyceraldehyde-3-phosphate dehydrogenase (GAPDH), phosphoglycerate kinase (PGK), pyruvate kinase (PK), alcohol dehydrogenase (ADH), pyruvate decarboxylase (PDC), and lactate dehydrogenase (LDH), were up-regulated in Xieyou 9308. The reaction catalyzed by PFK is the rate-limiting step of glycolysis; up-regulated PFK (Os05t0524400) may therefore reduce the limitation. In addition, three DG_HP_ (Os07t0181000, Os11t0148500, and Os12t0145700) that encode PK were up-regulated in Xieyou 9308. Interestingly, the expression of *OsPK1* (Os11t0148500) is stronger in adventitious roots than in the primary root, both of which serve as execution sites of absorption [41]. This suggests that Xieyou 9308 may have a stronger absorption capacity than its parental lines. Transcriptional expression levels of FBA (Os05t0402700, Os08t0120600, and Os11t0171300), which plays an important physiological role in accelerating cell growth and promoting root elongation [42], were up-regulated. In addition, transcriptional expression levels of LDH (Os02t0105400 and Os06t0104900), PDC (Os03t0293500, Os05t0469600, Os05t0469800, Os09t0371500, and Os10t0480900), and ADH (Os11t0210300, Os11t0210500, and Os11t0210600), which are involved in glycolysis pathways, were up-regulated. It has been suggested that the lactate initially produced by LDH lowers the pH, which in turn activates PDC and ADH [43,44]. LDH, PDC, and ADH transcripts may be involved in inducing the hypoxia pathway; their up-regulation in this study may be due to the anaerobic stress that the roots might have experienced in the hydroponic solution [45-47].

In contrast to animal cells, plant cells are enclosed by cell walls, which not only determine cell shape and provide structural support but also protect the plant against environmental stresses and regulate plant growth [48,49]. Cellulose is the most abundant biopolymer and the main structural component of plant cell walls. Our transcriptional profile analysis identified up-regulated transcripts that were related to cellulose synthesis, including cellulose synthase (CesA) (Os01t0750300, Os03t0808100, Os03t0837100, Os05t0176100, Os07t0208500, Os07t0208533, Os07t0252400, Os07t0424400, Os09t0422500, and Os10t0467800) and β-1, 4-endoglucanase (EGase) (Os03t0736300 and Os04t0497200). CesA up-regulation can promote root hair elongation, thus improving the absorption of water and nutrients by roots [50,51]. Interestingly, a previous study found that high expression of *OsGLU3* (Os04t0497200) can affect root cell wall cellulose synthesis and thus modulate root elongation and protect roots from environmental stresses [52].

### Complex regulation of plant hormone signal transduction

In multicellular organisms, cellular communication is important for coordinating the growth and differentiation of cells into new tissues and organs. As is well known, hormones act as signaling molecules in plants by mediating physiological responses. Similar to the results in studies by Kyndt *et al.*[15] and Wang *et al.*[32], our transcriptome analysis uncovered many DG_HP_ that were involved in the phytohormone response in root tissues. To illustrate, the abscisic acid (ABA) pathway is involved in the repression of lateral root development and adaption to environmental stresses [53,54]. In our study, many ABA-responsive transcripts exhibited different expression levels between the hybrid and its parents. For example, mRNA levels of four transcripts (Os03t0610900, Os04t0432000, Os07t0622000, and Os12t0586100) encoding SNF1-related protein kinase 2 (SnRK2), whose autophosphorylation is required for kinase activity towards downstream targets, were significantly more highly expressed in Xieyou 9308 than in its parents. In addition, similar to previous studies by Cohen *et al.*[55] and Santiago *et al.*[56], *PYR/PYL* ABA receptors (Os02t0255500 and Os10t0573400) were down-regulated, and type-2C protein phosphatase (PP2C, a negative regulator) (Os01t0583100 and Os05t0457200) was up-regulated. These results fit into the negative-feedback regulatory mechanism. Such re-setting of the ABA signaling pathway provides Xieyou 9308 with a dynamic mechanism for monitoring ABA levels and modulating ABA response [57].

Transcripts involved in the cytokinin (CTK) signaling pathway were also differentially expressed between the hybrid and its two parents in our study. The CTK pathway is involved in two aspects of root growth inhibition: the impedance of primary root elongation and the regulation of lateral root initiation [58]. A subset of CTK-responsive transcripts such as type-A response regulators (RRs-A) (Os01t0952500, Os02t0557800, Os04t0442300, and Os12t0139400) and type-B response regulators (RRs-B) (Os02t0796500, Os03t0224200, and Os06t0183100), showed significantly different expression patterns in root tissues. RRs-B are positive transcriptional regulators in the CTK signaling pathway, whereas RRs-A act as negative regulators [59]. The most likely model for this interaction is one in which RRs-A inhibit the activation of RRs-B by competing for phosphotransfer from upstream histidine phosphotransfer proteins; this model has been demonstrated in a few bacterial two-component systems [60-62]. Furthermore, previous studies have demonstrated that root elongation and lateral root formation in type-A mutants is more sensitive to CTK inhibition, and type-B mutants exhibit the opposite behavior [63-65]. The up-regulated RRs-A and down-regulated RRs-B observed in our study may thus work together to lessen the sensitivity of Xieyou 9308 root tissues to CTK inhibition.

### Comparison of significant DG_HP_ with QTLs for yield and root traits

In previous studies involving recombinant inbred lines and backcross populations derived from a cross between R9308 and Xieqingzao B, we identified a large number of QTLs for root traits, including root dry weight (RDW), root length (RL), root-shoot ratio (RS), total root length (TRL), root volume (RV), root surface area (RSA), root tip number (RTN), and root average diameter (RAD) [66-71]. The comparison of DG_HP_ with QTLs that were mapped consistently across years or environments revealed that 12 DG_HP_ in the carbohydrate metabolism pathway, encoding PFK, SUS, PDC, ADH, EGase, and CesA, and 2 DG_HP_ in the plant hormone signal transduction pathway, encoding RR-B and SnRK2, were located in root-related QTL regions (Table 6). As reported by Liang *et al.*[67], root traits, including RL, TRL, RSA, RV, RTN, and RDW, are positively correlated with yield-related traits such as grain yield per plant (GYD), number of panicles (NP), number of spikelets per panicle (NSP), and heading date (HD). We therefore investigated the potential association among the DG_HP_, QTLs for yield-related traits, and heterosis within many QTL regions, including associations between CesA (Os07t0208500, Os07t0208533, and Os07t0252400) and RM180–RM5436 for GYD, NP, NSP, and HD; CesA (Os03t0837100) and RM148–RM85 for HD; and PFK (Os05t0524400) and RM6972–RM274 for GYD. Interestingly, PFK, which is involved in glycolysis, was located in the interval RM6972–RM274 not only for RDW but also for GYD. In addition, three DG_HP_ encoding CesA were detected in the interval RM180–RM5436 on chromosome 7 for three different yield-related traits and six root-related traits (Table 6).

**Table 6:** Significant differentially expressed transcripts located in each of the QTL regions

**Trait**	**Chr.**	**Interval**	**Gene definition**	**RAP locus**
RL	3	RM282-RM6283	SUS	Os03t0340500, Os03t0401300
RDW	3	RM168-RM143	EGase	Os03t0736300
RL	4	RM5953-RM3317	SUS	Os04t0249500
RD	5	RM3800-RM3870	PP2C	Os05t0457200
RDW	5	RM3870-RM6972	PDC	Os05t0469600, Os05t0469800
RDW	5	RM6972-RM274	PFK	Os05t0524400
RV	6	RM6734-RM1163	RR-B	Os06t0183100
RL	7	RM234-RM118	SnRK2	Os07t0622000
RL, TRL, RSA, RV, RTN, RDW	7	RM180-RM5436	CesA	Os07t0208500, Os07t0208533, Os07t0252400
RFW	10	RM6100-RM3773	PDC	Os10t0480900
GYD, NP, NSP, HD	7	RM180-RM5436	CesA	Os07t0208500, Os07t0208533, Os07t0252400
HD	3	RM148-RM85	CesA	Os03t0837100
GYD	5	RM6972-RM274	PFK	Os05t0524400

In hybrids, diversity in gene expression can be the result of variation in *cis*-regulatory elements (e.g., promoter regions) or *trans*-regulatory elements (e.g., transcription factors) [72]. If differential expression of a gene is *cis*-induced, the gene may be located in a QTL region, whereas if it is *trans-*induced, a QTL may correspond with the *trans*-regulatory elements and be located distantly from the gene locus. In our DG_HP_ collections, we indeed found that some DG_HP_ encoded transcription factors, which may be involved in *trans*-regulation, in the QTL regions (Additional file 9: Table S6). The expression of candidate transcripts in these QTL regions may serve as a starting point to understand the molecular mechanisms underlying heterosis. The application of these results is expected to provide an opportunity to breed elite rice varieties that process both yield and root heterosis.

## Conclusion

In this study, we used RNA-Seq to systematically investigate the global transcriptomes of roots from the super-hybrid rice Xieyou 9308 and its parents at tillering and heading stages, generating a useful resource for the rice community. We analyzed DG_HP_ and compared them with QTLs for yield and root traits, providing clues for candidate transcripts that may significantly contribute to root development and yield production. The changes in the expression of candidate transcripts may provide valuable information for future studies on molecular mechanisms underlying root heterosis.

## Methods

### Plant materials and growing conditions

Xieyou 9308, a super-hybrid rice variety commonly planted in China, and its parents Xieqingzao B (female) and R9308 (male) were used in this study. All experiments were conducted in 2011. Rice seeds were surface-sterilized with 3% H_2_O_2_ for 10 min and then rinsed several times with distilled water. After soaking in distilled water at 37°C for 2 d, germinated seeds were sown in the field at the China National Rice Research Institute, Fuyang, China. After approximately 30 d, 40 seedlings of each genotype were transplanted into plots of plastic foam floating in a pool filled with nutrient solution. Roots were sampled with ten replicates at tillering and heading stages to measure root traits and estimate heterosis. In addition, two roots of every genotype at each stage were collected and stored at −80°C in preparation for RNA-Seq analysis.

### Root heterosis measurements

To determine dry weight and root-shoot ratio, shoots and root systems were placed in an oven set at 110°C for 80 min, followed by drying at 80°C for 4 d. Root length was measured manually. MPH and HPH were calculated according to the following formulas: MPH = (F_1_ − MP)/MP in *%* and HPH = (F_1_ − HP)/HP in *%*, where F_1_ is the performance of the hybrid, MP is the average performance of the two parents, and HP is the best performance of the two parents. Hypothesis testing was performed using *t*-test.

### RNA extraction, cDNA library preparation, and sequencing

Total RNA was extracted from roots using Trizol reagent (Invitrogen, Carlsbad, CA, USA) and purified using RNeasy Plant Mini Kit (Qiagen, Valencia, CA). The RNA quality was checked on a Bioanalyzer 2100 (Aligent, Santa Clara, CA); RNA Integrity Number (RIN) values were greater than 8.5 for all samples. Sequencing libraries were prepared according to the manufacturer’s instructions (Illumina, San Diego, CA). Poly-A-containing mRNA was isolated from the total RNA, subjected to two purification rounds using poly-T oligo-attached magnetic beads, and fragmented using an RNA fragmentation kit. First strand cDNA was generated using reverse transcriptase and random primers. Following the second strand cDNA synthesis and adaptor ligation, 200-bp cDNA fragments were isolated using gel electrophoresis and amplified by 18 cycles of PCR. The products were loaded onto an Illumina HiSeq2000 instrument and subjected to 100 cycles of paired-end (2 × 100 bp) sequencing. The processing of fluorescent images into sequences, base-calling and quality value calculations were performed using the Illumina data processing pipeline (version 1.8). The sequence reads were submitted to GenBank GEO database under accession number GSE41797 (http://www.ncbi.nlm.nih.gov/geo/query/acc.cgi?acc=GSE41797).

### Mapping of short reads and assessment of differential gene expression

Raw reads were filtered to obtain high-quality reads by removing low-quality reads containing more than 30% bases with Q < 20. After trimming low-quality bases (Q < 20) from the 5' and 3' ends of the remaining reads, the resulting high-quality reads were mapped onto the Nipponbare reference genome (IRGSP build 5.0) using RSEM (v1.1.11) [73]. Differential expression was estimated and tested with the software package edgeR (R version: 2.14, edgeR version: 2.3.52) [74]; we quantified gene expression levels in terms of RPKM [75], calculated FDR, and estimated FC and log_2_ values of FC. Transcripts that exhibited an FDR ≤ 0.05 and an estimated absolute log_2_ (FC) ≥ 1 were determined to be significantly differentially expressed. The transcript coverage was calculated as the number of mapped reads in a locus multiplied by 100 bp and then divided by the summed exon length of the locus.

For statistical analysis, we used the following analysis of variance (ANOVA) model: y = u + (GA) + (GD) + (SR) + e, where y is the acquired gene expression, u is the overall mean, GA is the additive effect, GD is the dominant effect, SR is the replication effect, and e is the residual error. The *d*/*a* ratio, also referred to as dominance ratio or potence, *h*_p_[76] (where *a* and *d* represent GA and GD, respectively [77,78]), was calculated to measure the nonadditivity of the F_1_ hybrid relative to the parents. To avoid troublesome statistical properties, Vuylsteke *et al.*[79] proposed using *d* - *a* (*d* > 0) or *d* + *a* (*d* < 0) instead of *d*/*a*. We accordingly constructed 99.8% confidence intervals for *d* - *a* (*d* > 0) and *d* + *a* (*d* < 0). If *d* > 0 and the confidence interval constructed for *d* - *a* included zero, then *h*_p_ = 1. When the confidence interval did not include zero and was positive, then *h*_p_ > 1. An analogous procedure was applied for *d* + *a* (*d* < 0).

### Cluster analysis

Cluster analysis was carried out for all annotated transcripts from the hybrid Xieyou 9308 and its parents at tillering and heading stages. The RPKM-normalized expression counts for each transcript were clustered with the software Cluster 3.0, and the results were visualized using Treeview [80].

### Validation of RNA-Seq by qRT-PCR

Total RNA from two sequenced samples and two new samples was treated with DNase, and first-strand cDNA was generated using a RevertAid First Strand cDNA Synthesis kit (Fermentas, Vilnius, Lithuania). SYBR-based qRT-PCR reactions (SYBR Green I, Osaka, Japan) were performed on a LightCycler 480 system (Roche, Basel, Switzerland) using the following reaction conditions: 95°C for 1 min followed by 40 cycles of 95°C for 10 s and 60°C for 30 s. All qRT-PCR reactions were performed in triplicate, and the results were analyzed with the system’s relative quantification software (ver.1.5) based on the delta-delta-Ct method (Roche). The detection threshold cycle for each reaction was normalized against the expression level of the rice *Actin1* gene with primer sequences 5'-TGGCATCTCTCAGCACATTCC-3' and 5'-TGCACAATGGATGGGTCAGA-3'.

## Abbreviations

DEGs: Differentially expressed genes; DG_HP_: Differentially expressed genes between the hybrid and its parents; QTL: Quantitative trait locus; EST: Expressed sequence tag; SAGE: Serial analysis of gene expression; RNA-Seq: RNA sequencing technology; MPH: Mid-parent heterosis; HPH: Best-parent heterosis; AHP: Above high-parent; HPL: High-parent level; MPL: Mid-parent level; LPL: Low-parent level; BLP: Below low-parent; GO: Gene Ontology; WEGO: Web Gene Ontology Annotation Plot; KEGG: Kyoto Encyclopedia of Genes and Genomes; qRT-PCR: Quantitative real-time PCR; RAP-DB: Rice Annotation Project Database; SUS: Sucrose synthase; PFK: Phosphofructose kinase; FBA: Fructose-bisphosphate aldolase; GAPDH: Glyceraldehyde-3-phosphate dehydrogenase; PGK: Phosphoglycerate kinase; PK: Pyruvate kinase; ADH: Alcohol dehydrogenase; PDC: Pyruvate decarboxylase; LDH: Lactate dehydrogenase; CesA: Cellulose synthase; EGase: β-1,4-endoglucanase; SnRK2: SNF1-related protein kinase 2; PP2C: Type-2C protein phosphatase; ABA: Abscisic acid; CTK: Cytokinin; RRs-A: Type-A response regulators; RRs-B: Type-B response regulators; RDW: Root dry weight; RL: Root length; RS: Root-shoot ratio; TRL: Total root length; RV: Root volume; RSA: Root surface area; RTN: Root tip number; RAD: Root average diameter; GYD: Grain yield per plant; NP: Number of panicles; NSP: Number of spikelets per panicle; HD: Heading date.

## Competing interests

The authors declare that they have no competing interests.

## Authors’ contributions

RZ, YF, LC and SC conceived and designed the experiments. RZ, HW, GD and ZY collected heterosis phenotype data and prepared RNA. RZ and YZ performed data processing and qRT-PCR. RZ and YF contributed to the interpretation of the data and drafted the manuscript. XZ, WW, DC, and XS helped to revise the manuscript. LC and SC supervised the study and revised the manuscript. All authors read and approved the final manuscript.

## Supplementary Material

Additional file 1 **Figure S1.** Scatterplots comparing gene expression scores from biological replicates of Xieyou 9308 and the two parents. Numbers12 and 34 denote biological replicates at tillering and heading stages, respectively. R, X, and F refer to R9308, Xieqingzao B, and Xieyou 9308, respectively. Click here for file

Additional file 2 **Figure S2.** Comparison of the root dry weight of Xieyou 9308, Xieqingzao B, and R9308 at tillering and heading stages. Click here for file

Additional file 3 **Table S1.** RPKM of RAP2-annotated transcripts at tillering and heading stages. Click here for file

Additional file 4 **Table S2.** Classification of DG_HP_ based on the dominance ratio *h*_p_. Click here for file

Additional file 5 **Figure S3.** The number of DG_HP_ in the biological process category at tillering and heading stages. Click here for file

Additional file 6 **Tables S3 and Table S4.** Significant KO terms of DG_HP_ at the tillering stage (Table S3) and the heading stage (Table S4). Click here for file

Additional file 7 **Figure S4.** The number of DG_HP_ in each KEGG pathway at tillering and heading stages. Click here for file

Additional file 8 **Table S5.** Experimental validation using qRT-PCR and primer sequences for qRT-PCR expression analysis. Click here for file

Additional file 9 **Table S6.** Significant differentially expressed *trans*-regulatory elements located in each of the QTL regions. Click here for file
