# Supplementary figures and images for: Transcriptome analysis of rice root heterosis by RNA-Seq
Source: BMC Genomics. 2013 Jan 16;14:19. doi: 10.1186/1471-2164-14-19 (PMC3556317; doi:10.1186/1471-2164-14-19)

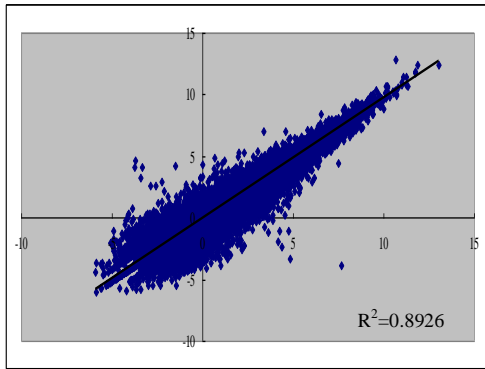

**R12**

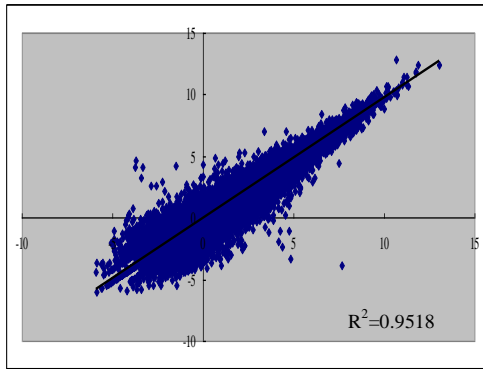

**R34**

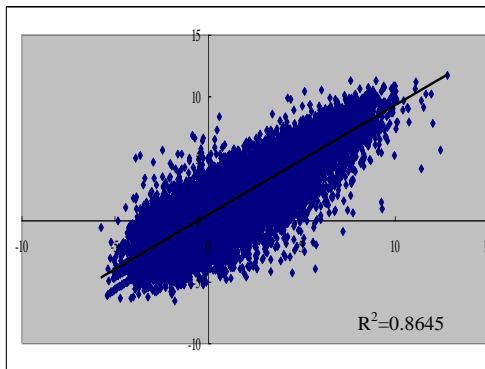

**F12**

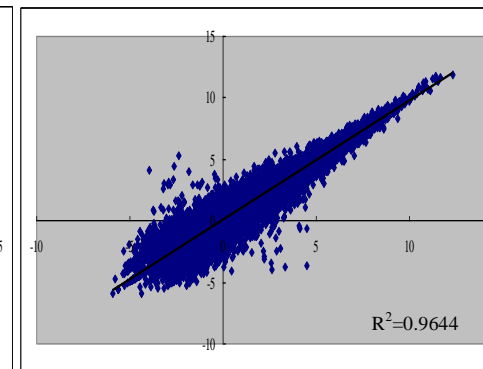

**F34**

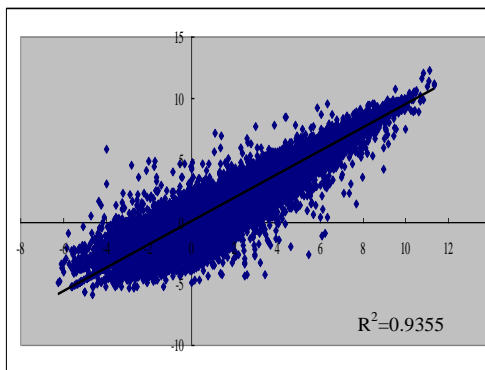

**X12**

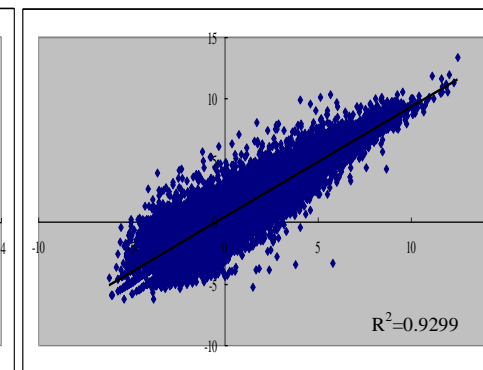

**X34**

Supplement: Additional file 1 — Figure S1. Scatterplots comparing gene expression scores from biological replicates of Xieyou 9308 and the two parents. Numbers12 and 34 denote biological replicates at tillering and heading stages, respectively. R, X, and F refer to R9308, Xieqingzao B, and Xieyou 9308, respectively. [file 1471-2164-14-19-S1.pdf]

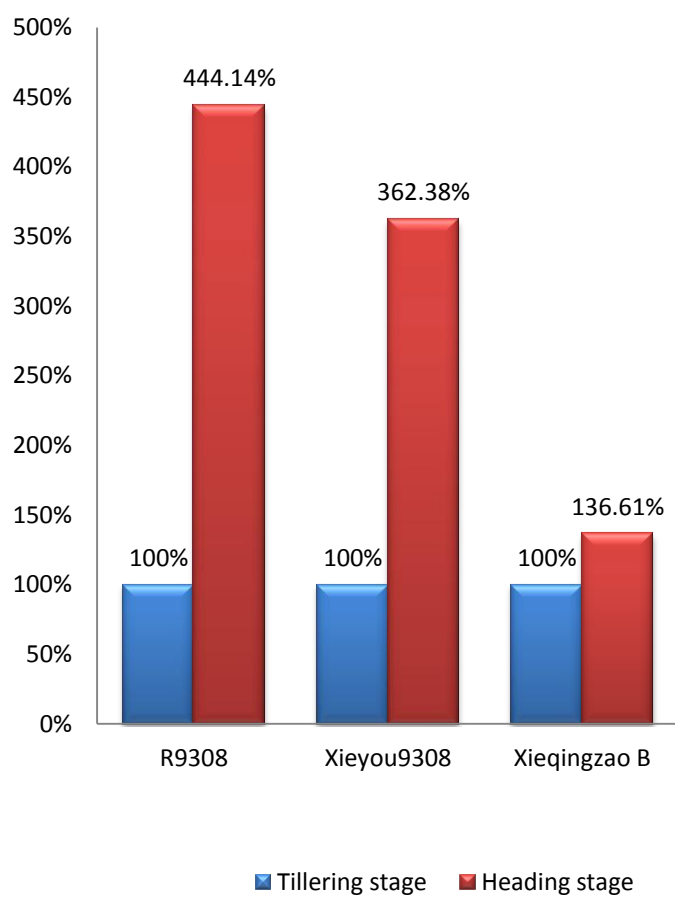

Supplement: Additional file 2 — Figure S2. Comparison of the root dry weight of Xieyou 9308, Xieqingzao B, and R9308 at tillering and heading stages. [file 1471-2164-14-19-S2.pdf]

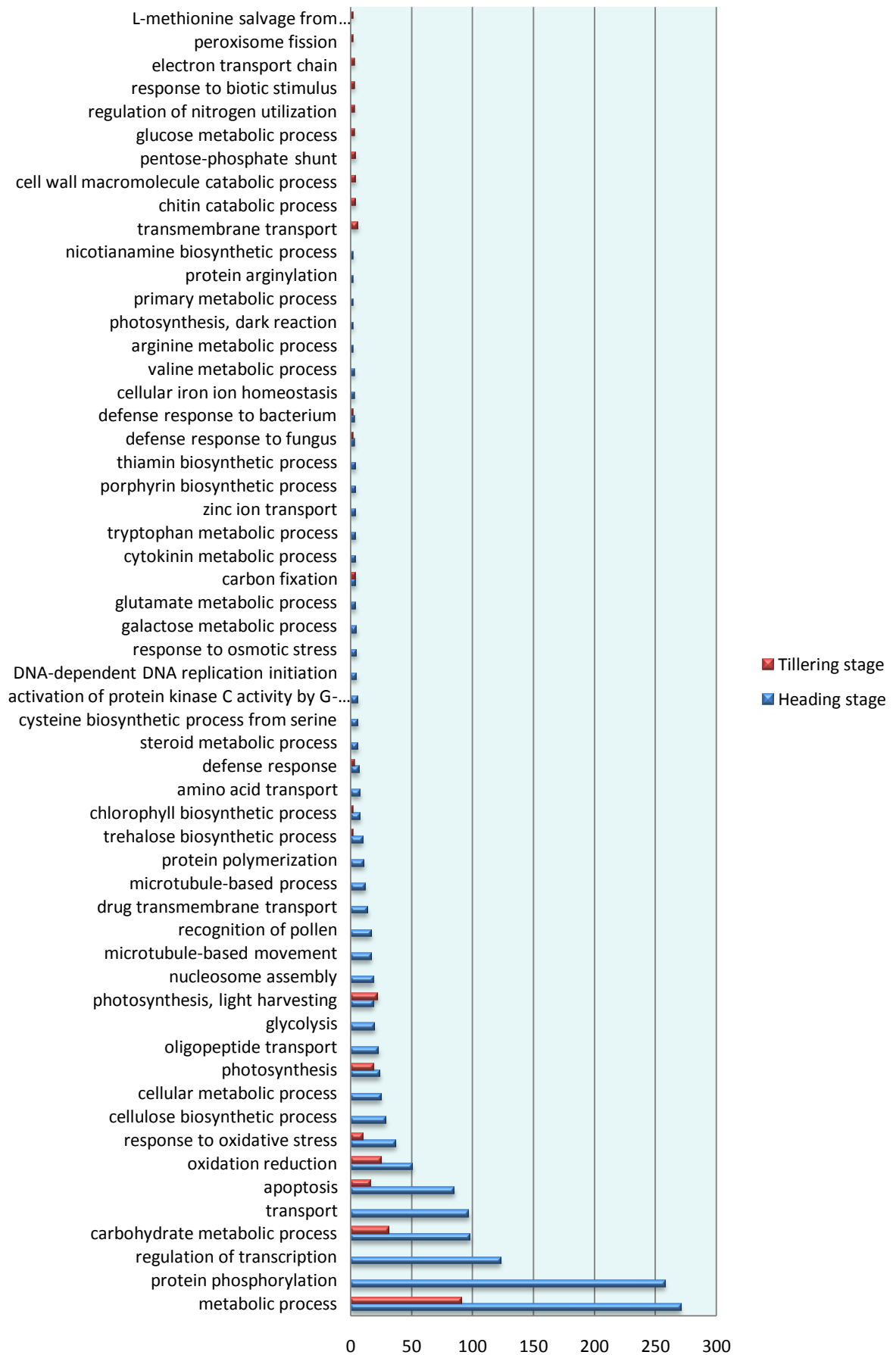

Supplement: Additional file 5 — Figure S3. The number of DGHP in the biological process category at tillering and heading stages. [file 1471-2164-14-19-S5.pdf]

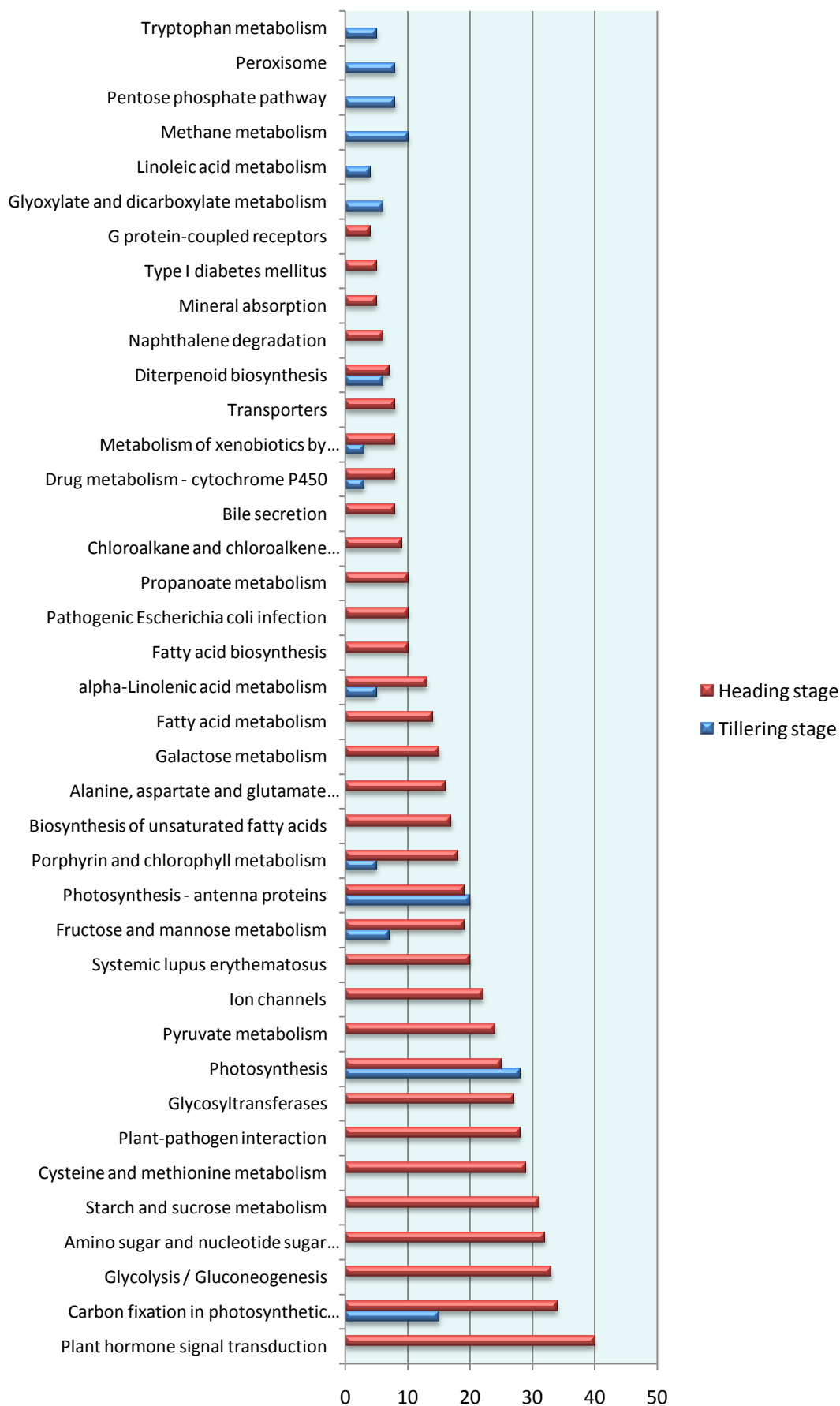

Supplement: Additional file 7 — Figure S4. The number of DGHP in each KEGG pathway at tillering and heading stages. [file 1471-2164-14-19-S7.pdf]
