# Supplementary material for: Transcriptome analysis of rice root heterosis by RNA-Seq
Source: BMC Genomics. 2013 Jan 16;14:19. doi: 10.1186/1471-2164-14-19 (PMC3556317; doi:10.1186/1471-2164-14-19)
Supplement: Additional file 6 — Tables S3 and Table S4. Significant KO terms of DGHP at the tillering stage (Table S3) and the heading stage (Table S4). [file 1471-2164-14-19-S6.doc]

**Table S3** **Significant ko terms of DG_HP_ at tillering stage (*P*-value < 0.05)**

| **ko_term** | ko_annotation | *P*-value |
| --- | --- | --- |
| **ko00195** | Photosynthesis | 1.39E-23 |
| **ko00196** | Photosynthesis - antenna proteins | 2.03E-21 |
| **ko00710** | Carbon fixation in photosynthetic organisms | 3.71E-05 |
| **ko00904** | Diterpenoid biosynthesis | 0.000111 |
| **ko00680** | Methane metabolism | 0.001902 |
| **ko00030** | Pentose phosphate pathway | 0.002055 |
| **ko00630** | Glyoxylate and dicarboxylate metabolism | 0.008142 |
| **ko00380** | Tryptophan metabolism | 0.011374 |
| **ko00591** | Linoleic acid metabolism | 0.012365 |
| **ko00051** | Fructose and mannose metabolism | 0.021504 |
| **ko04146** | Peroxisome | 0.021975 |
| **ko00982** | Drug metabolism - cytochrome P450 | 0.036718 |
| **ko00980** | Metabolism of xenobiotics by cytochrome P450 | 0.036718 |
| **ko00592** | alpha-Linolenic acid metabolism | 0.037322 |
| **ko00860** | Porphyrin and chlorophyll metabolism | 0.046416 |

**Table S4 Significant ko terms of DG_HP_ at heading stage (*P***-value < 0.05)

| ko_term | ko_annotation | *P*-value |
| --- | --- | --- |
| ko04030 | G protein-coupled receptors | 0.029421 |
| ko04978 | Mineral absorption | 0.003032 |
| ko04940 | Type I diabetes mellitus | 0.022162 |
| ko00626 | Naphthalene degradation | 0.012399 |
| ko00904 | Diterpenoid biosynthesis | 0.0351 |
| ko00980 | Metabolism of xenobiotics by cytochrome P450 | 0.006893 |
| ko00982 | Drug metabolism - cytochrome P450 | 0.006893 |
| ko04976 | Bile secretion | 0.020778 |
| ko02000 | Transporters | 0.047843 |
| ko00625 | Chloroalkane and chloroalkene degradation | 0.003962 |
| ko00640 | Propanoate metabolism | 0.018122 |
| ko00061 | Fatty acid biosynthesis | 0.024782 |
| ko05130 | Pathogenic Escherichia coli infection | 0.048767 |
| ko00592 | alpha-Linolenic acid metabolism | 0.031301 |
| ko00071 | Fatty acid metabolism | 0.029152 |
| ko00052 | Galactose metabolism | 0.044741 |
| ko00250 | Alanine, aspartate and glutamate metabolism | 0.001883 |
| ko01040 | Biosynthesis of unsaturated fatty acids | 0.000795 |
| ko00860 | Porphyrin and chlorophyll metabolism | 0.000582 |
| ko00196 | Photosynthesis - antenna proteins | 8.28E-09 |
| ko00051 | Fructose and mannose metabolism | 0.014675 |
| ko05322 | Systemic lupus erythematosus | 0.000313 |
| ko04040 | Ion channels | 6.04E-07 |
| ko00620 | Pyruvate metabolism | 0.018842 |
| ko00195 | Photosynthesis | 2.69E-06 |
| ko01003 | Glycosyltransferases | 2.04E-05 |
| ko04626 | Plant-pathogen interaction | 0.000858 |
| ko00270 | Cysteine and methionine metabolism | 0.000639 |
| ko00500 | Starch and sucrose metabolism | 0.023338 |
| ko00520 | Amino sugar and nucleotide sugar metabolism | 0.000491 |
| ko00010 | Glycolysis / Gluconeogenesis | 0.027157 |
| ko00710 | Carbon fixation in photosynthetic organisms | 0.000165 |
| ko04075 | Plant hormone signal transduction | 0.004102 |
